# A New Spectral Shift-Based Method to Characterize Molecular Interactions

**DOI:** 10.1089/adt.2021.133

**Published:** 2022-03-08

**Authors:** Andreas Langer, Tanja Bartoschik, Ondrej Cehlar, Stefan Duhr, Philipp Baaske, Werner Streicher

**Affiliations:** ^1^ NanoTemper Technologies GmbH, Munich, Germany.; ^2^ Institute of Neuroimmunology, Slovak Academy of Sciences, Bratislava, Slovakia.

**Keywords:** biomolecular interaction, binding studies, interaction analysis, spectral shift, thermodynamics

## Abstract

There are many fluorescence-based applications that can be used to characterize molecular interactions. However, available methods often depend on site-specific labeling techniques or binding-induced changes in conformation or size of the probed target molecule. To overcome these limitations, we applied a ratiometric dual-emission approach that quantifies ligand-induced spectral shifts with sub-nanometer sensitivity. The use of environment-sensitive near-infrared dyes with the method we describe enables affinity measurements and thermodynamic characterization without the explicit need for site-specific labeling or ligand-induced conformational changes. We demonstrate that in-solution spectral shift measurements enable precise characterization of molecular interactions for a variety of biomolecules, including proteins, antibodies, and nucleic acids. Thereby, the described method is not limited to a subset of molecules since even the most challenging samples of research and drug discovery projects like membrane proteins and intrinsically disordered proteins can be analyzed.

## Introduction

Fluorescence-based technologies are commonly used in drug discovery to identify new molecules with therapeutic potential.^1–3^ With the number of compound libraries steadily increasing and new challenging targets emerging from genetic and proteomic research, ligand binding assays based on fluorescence intensity changes,^4^ resonance energy transfer (*i.e.,* fluorescence resonance energy transfer [FRET], bioluminescence resonance energy transfer [BRET], time-resolved fluorescence energy transfer),^5–7^ or fluorescence polarization/anisotropy (FP)^8,9^ continue to be a popular choice. Not only do they offer a highly sensitive readout, but they are also relatively simple, robust, scalable, and cost effective.

Methods based on fluorescence can traditionally be classified into two categories: Those in which only one of the interacting molecules needs to be fluorescently active like FP, and those in which energy is transferred from one molecule to another to derive binding information, such as BRET or FRET assays. The main drawback of methods from the latter category is that it is rare that both molecules involved in an interaction are intrinsically fluorescent or easily modified with a fluorophore or quencher. Therefore, measurements generally require more planning and optimization, for example, by designing suitable FRET pairs, site-specific modifications or by developing sophisticated competition assays with potentially difficult-to-produce fluorescent tool compounds.

In FP assays, only one binding partner has to be fluorescent. Despite this advantage, the required change in molecular size upon binding makes this method less suited for characterizing interactions between two large molecules or those in which only the larger of the two interaction partners can be labeled.^8^ A simpler alternative is measuring fluorescence intensity changes upon ligand binding. Intrinsic tryptophan fluorescence quenching assays have been found to work well for proteins and hydrophobic ligands based on changes in the local environment of the tryptophan residues as a consequence of molecular interaction.^10,11^

In scenarios where the ligands themselves fluoresce, or the intrinsic protein fluorescence is not sufficiently altered by ligand binding, one may consider covalently attaching an extrinsic dye. Environment-sensitive dyes are particularly useful in these situations. Small alterations in their microenvironment change their fluorescent properties, for example, by transient interactions with certain amino acids of a protein or nucleobases of a nucleic acid.^12,13^ An additional advantage of using extrinsic dyes is that they are generally brighter than intrinsic fluorophores, thus reducing the required target concentrations in an assay. Using less target not only lowers sample consumption but also enables the determination of picomolar binding affinities.^14^

Relying simply on intensity changes upon ligand binding, however, is often prone to error. Pitfalls of intensity-only measurements are false positives or false negatives due to intrinsic ligand fluorescence or non-specific effects, such as binding of labeled molecules to labware. In addition, very small intensity changes can remain masked behind pipetting errors or measurement noise. Many of these limitations can be circumvented by ratiometric fluorescence measurements, which, due to their nature, do not depend on absolute fluorescence intensities.

Small molecule-based ratiometric fluorescence probes are available for many cations, anions, and biomolecules and are commonly used for concentration measurements of chemical species.^15^ Some examples include dyes for the detection of chemical substances such as sulfur dioxide^16^ or calcium ions.^17^ Stokes shifts of these optimized probes tend to be rather large, sometimes even above one hundred nanometers. Furthermore, wavelength shifts in the emission spectrum of selected dyes were used in imaging and microscopy applications to measure membrane potentials^18^ and pH changes^19^ in living cells.

One explanation of the environment-sensitivity of dyes is cis-trans-isomerization.^20^ In their ground state, dyes can exist as *cis* or *trans* isomers, which usually differ in their spectroscopic properties (*e.g.,* fluorescence lifetime, quantum yield, and maximum wavelength of absorbance). The cis-isomer of Cy3, for example, is red-shifted by ∼20 nm.^21,22^ Changes in the microenvironment of a dye can affect the dye's rotational freedom and lead to shifts of the steady-state population density between *cis* and *trans* isomers. An extrinsic dye attached to a protein may therefore experience a spectral shift when a ligand binds in its proximity, or when a conformational change leads to an alteration of the local viscosity.

Shifts in absorption and emission wavelength can also occur in response to changes in hydrogen-bonding or polarity. Bathochromic shifts are usually related to a more solvent-exposed environment.^23^ For example, when coupled to biotin, red-absorbing dyes such as Cy5 or Alexa647 have been found to undergo strong bathochromic shifts upon binding to streptavidin.^24^

It has also been reported that dyes shift their fluorescence emission when conjugated to IgG.^25^ By site-specific labeling at positions sensitive to conformational changes, allosteric binders of several kinases could be identified through ratiometric measurements.^26–28^ Red dyes are particularly well suited to be used as covalently attached extrinsic fluorophores on proteins in small molecule screening campaigns. They reduce interference from intrinsic compound fluorescence, which mainly occurs at lower wavelengths, and also experience lower background signals from dust.^27^

In these applications, ratiometric fluorescence measurements are typically performed with standard spectrofluorometers or fluorescence plate-readers that may fail to resolve very small spectral shifts. Therefore, the success of a binding assay requires sufficient knowledge of the system to ensure that the site-specific labeling of the target is at a site that is sensitive enough to detect conformational changes upon binding. In addition, measurements in cuvettes usually require large amounts of material, thus limiting the general use of this approach.

Here, we present a highly sensitive method to characterize a broad range of biomolecular interactions by isothermal spectral shift analysis using a dedicated dual-emission optical system. Instead of measuring the full emission spectrum, the fluorescence is recorded simultaneously only at two pre-selected wavelengths with photon-multiplier-tubes, which greatly enhances sensitivity. Furthermore, the method does not require site-specific labeling or conformational changes of the target molecule upon binding and only uses a few microliters of sample at low nanomolar concentration per data point.

## Materials and Methods

### Materials

Carbonic Anhydrase II from bovine erythrocytes (bCA-II, cat# C2522) and bCA-II inhibitors acetazolamide, furosemide, sulfanilamide, and benzenesulfonamide were purchased from Sigma Aldrich. His_9_-tagged glucose transporter 1 (GLUT1) was obtained from Crelux GmbH (Martinsried, Germany). GLUT1 inhibitor BAY-876 was purchased from Sigma Aldrich. Anti-Tau antibody Tau5 was purchased from Invitrogen, Thermo Fisher Scientific.

Tau isoforms 2N4R and 0N3R and shorter fragments tau_221–441_ and tau_343–441_ were expressed and purified according to previously published procedure.^29^ Briefly, a two-step cation exchange chromatography was used, followed by a size exclusion step, and desalting into phosphate-buffered saline (PBS) supplemented with argon. The concentration of tau proteins was determined from the absorbance at 205 nm, with absorption coefficients calculated from the protein sequence.^30^

Chemically synthesized DNA oligonucleotides were obtained from Metabion GmbH (Planegg, Germany). A Cy5-labeled 13-mer including a short poly-T spacer in front of a 10 base-long recognition sequence (sequence 5′ Cy5 TTT GGA CTT CAG G 3′) was designed as target and a complementary 10-mer (sequence 3′ CCT GAA GTC C 5′) as ligand. All other chemicals and reagents used in this study were of the highest available analytic grade.

### Dual-Emission Spectral Shift Measurements

All dual-emission spectral shift measurements in this work were performed in coated capillaries (cat# MO-K025; NanoTemper Technologies GmbH) using the Monolith X instrument equipped with dual-emission detection optics (NanoTemper Technologies GmbH). The required volume per data point was 10 μL. Environment-sensitive near-infrared dyes with an emission maximum at around 660 nm (*e.g.,* RED 2nd Generation dyes, NanoTemper Technologies GmbH, or Cy5) were used as fluorescent reporters. For each ratiometric reading, the fluorescence was recorded simultaneously at 650 and 670 nm for 5 s (*Table 1*).

**Table 1.** Dual-Emission Spectral Shift Measurements

Step	Parameter	Value	Description
1	Fluorescent labeling of target	∼45 min	Dye with emission maximum at ∼660 nm
2	Dilution of ligand	10 μL	16-point serial dilution
3	Adding target to ligand dilution series	10 μL	Mixing by pipetting up and down
4	Preincubation	∼5–30 min	At room temperature with light protection
5	Loading of samples into capillaries and device	16	MO-K025 capillaries, Monolith X device
6	Spectral shift measurement	5 min	Auto-excitation, 5 s reading per capillary
7	Data analysis	1 min	K_d_ analysis in MO.Control software

Step Notes: (1) Labeling of nucleic acids is ideally performed during synthesis. Proteins can, for example, be labeled with covalent lysine- or cysteine-reactive dyes or with dyes that bind non-covalently to the protein's His-tag or SNAP-Tag^®^.

(2) A 16-point serial dilution of the ligand is prepared at a final volume of 10 μL in each dilution step. The highest ligand concentration is set to ∼20 × above the expected K_d_.

(3) 10 μL of fluorescently labeled target is added to the ligand dilution series and samples are mixed by pipetting up and down. Depending on the K_d_ of the interaction, the final assay concentration of the target is typically between 5 and 20 nM.

(4) To ensure equilibrium, the dilution series is incubated at room temperature in the dark. For interactions with high affinities, longer incubation times may be needed to reach equilibrium.

(5) A volume of 10 μL from each tube of the dilution series is filled into capillaries and loaded into the Monolith X device.

(6) Each capillary is measured for 5 s, and fluorescence intensity values are recorded at 650 and 670 nm.

(7) 670 nm/650 nm ratios are calculated automatically in the control software, and data are fitted with a 1:1 binding model to derive the K_d_ value.

### Protein Labeling

Covalent labeling of lysine residues of bCA-II was performed using the Protein Labeling Kit RED-NHS 2nd Generation (cat# MO-L011; NanoTemper Technologies GmbH). In brief, bCA-II was prepared at a final concentration of 20 μM and supplemented with an equimolar concentration of ZnCl_2_. A threefold molar excess of dye was added to the protein, followed by incubation for 30 min at room temperature in the dark. Unreacted dye was removed using the B-Column of the labeling kit. Protein concentration (3.2 μM) and degree-of-labeling (0.9) were measured with a Nanodrop.

The GLUT1 His-tag was labeled using the His-Tag Labeling Kit RED-tris-NTA 2nd Generation (cat# MO-L018; NanoTemper Technologies GmbH). GLUT1 was diluted in assay buffer (PBS, pH 7.4, 0.05% DDM) to a working concentration of 2 μM. For affinity determination between His-tag and tris-NTA dye, a 16-step serial dilution of GLUT1 was prepared with 10 μL in each sample. Next, 10 μL of 10 nM dye dissolved in assay buffer was added to all vials of the serial dilution. The samples were mixed by pipetting up and down and then incubated for 30 min at room temperature in the dark, before samples were loaded into capillaries and into the Monolith X device. Based on the measured binding affinity between His-tag and tris-NTA dye, a 200 μL solution of labeled GLUT1 was prepared by mixing 100 μL of 80 nM GLUT1 with 100 μL of 20 nM dye.

Covalent labeling of lysine residues of Tau5 antibody was performed using the Protein Labeling Kit RED-NHS 2nd Generation (cat# MO-L011; NanoTemper Technologies GmbH). In brief, 50 μL of a 3.3 μM antibody solution was mixed with a threefold molar excess of dye and incubated for 30 min at room temperature in the dark. Unreacted dye was removed using the B-Column of the labeling kit. Protein concentration (0.3 μM) and degree-of-labeling (0.7) were measured with a Nanodrop.

### Protein–Small Molecule Interactions

bCA-II inhibitors acetazolamide, furosemide, sulfanilamide, and benzenesulfonamide were prepared as 10 mM stocks in dimethyl sulfoxide (DMSO). Four microliters of each 10 mM inhibitor were mixed with 96 μL of assay buffer (50 mM Tris-HCl, pH 7.8, 150 mM NaCl, 10 mM MgCl_2_, 0.05% Pluronic^®^ F-127) to obtain 400 μM inhibitor working solutions. Any further dilution steps were carried out in assay buffer supplemented with 4% DMSO.

Due to the different expected affinities, the highest final concentrations in the 16-point dilution series were 5 μM for acetazolamide, 50 μM for furosemide and benzenesulfonamide, and 200 μM for sulfanilamide. The final concentration of labeled bCA-II in the assays was 20 nM and the final DMSO concentration was 2%. After 5 min of incubation, samples were loaded into capillaries and placed into the device. Spectral shift measurements were performed at 25°C.

### Membrane Protein–Ligand Interactions

The GLUT1 inhibitor BAY-876 was prepared as 1 mM DMSO stock and subsequently diluted in assay buffer (PBS, pH 7.4, 0.05% DDM) to a highest final concentration of 1 μM. This solution was used to prepare a 16-step dilution series in assay buffer with 10 μL in each sample. Next, 10 μL of a solution containing 40 nM GLUT1 mixed with 10 nM dye was added to all vials of the serial dilution. The samples were mixed by pipetting up and down and then incubated for 30 min at room temperature in the dark to assure sufficient equilibration before spectral shift measurements were carried out at 25°C. As a control experiment, the measurement was repeated for a dilution series of BAY-876 and 10 nM dye without His-tagged GLUT1.

### Antibody–Protein Interactions

Tau proteins were prediluted in assay buffer (PBS, pH 7.4, supplemented with 0.01% Pluronic F-127) to working concentrations of 10 μM. The highest final ligand concentration in each 16-point dilution series was 5 μM and the final concentration of labeled Tau5 antibody was 4 nM. Each dilution series was incubated for at least 30 min before loading into capillaries and the Monolith X device. Spectral shift measurements were performed at 25°C.

### Thermodynamics of DNA Hybridization

Lyophilized DNA oligonucleotides were resuspended in ddH_2_O to a concentration of 100 μM. Assay buffer (20 mM sodium phosphate buffer, pH 7.2, 100 mM NaCl, 0.05% TWEEN^®^ 20) was prepared as twofold stock. Cy5-labeled 13-mer was further diluted in twofold assay buffer. Serial dilution of 10-mer was performed in ddH_2_O. The highest final concentration of 10-mer in the 16-point dilution series was 2 μM and the final concentration of Cy5-labeled 13-mer was 2.5 nM with 20 μL in each sample. For ratiometric fluorescence analysis, the device was adjusted to the desired temperature and samples were incubated for 5 min before starting the measurement. Measurements were performed at 23°C to 37°C in 2°C increments and then repeated in reverse order. Capillaries were sealed with Capillary Sealing Paste (cat# PR-P001; NanoTemper Technologies GmbH) to prevent evaporation.

### Full Spectrum Measurements

Full emission spectrum measurements were performed on a JASCO FP-8300 Fluorescence Spectrometer (JASCO Deutschland GmbH, Pfungstadt, Germany) in a High Precision Cell glass cuvette (cat# 101-10-40; Hellma Analytics, Jena, Germany), using an excitation wavelength of 600 nm, a scan speed of 20 nm/min and excitation and emission bandwidths of 5 and 2.5 nm, respectively. The volume was 500 μL and the concentrations used were 1 μM Cy5-labeled 13-mer (2 μM unlabeled 10-mer) and 20 nM labeled bCA-II (1 μM acetazolamide).

### Fluorescence Microplate Reader Measurements

Fluorescence measurements were performed on a CLARIOstar fluorescence microplate reader (BMG Labtech GmbH, Ortenberg, Germany) in a Greiner 384-well plate with wavelength settings of 590–20 (excitation), 640–20 (emission 1), and 680–20 (emission 2). The volume per well was 100 μL, the highest final concentration of acetazolamide in the 16-point dilution series was 5 μM and the final concentration of labeled bCA-II was 20 nM.

### Data Analysis

Ratios of the fluorescence intensities at 670 and 650 nm were calculated automatically within the MO.Control software (NanoTemper Technologies GmbH). For K_d_ determination, dose–response curves were fitted with a binding model that describes a molecular interaction with a 1:1 stoichiometry according to the law of mass action. In this model, the fraction bound f(c_ligand_) at a given ligand concentration c_ligand_ in equilibrium is given as

(1) $$\begin{array}{c} f\left(c_{ligand}\right)\\ =\frac{c_{ligand}+c_{target}+K_{d}-\sqrt{{\left(c_{ligand}+c_{target}+K_{d}\right)}^{2}-4\cdot c_{ligand}\cdot c_{target}}}{2c_{target}} \end{array}$$

where K_d_ is the dissociation constant, or binding affinity; c_target_ is the final concentration of target in the assay.

The K_d_ is then estimated by fitting the equation

(2) $$R\left(c_{ligand}\right)=R_{unbound}+f\left(c_{ligand}\right)\cdot \left(R_{bound}-R_{unbound}\right)$$

where R(c_ligand_) is the 670 nm/650 nm ratio value at a given ligand concentration c_ligand_; R_unbound_ is the ratio value of the target alone; R_bound_ is the ratio value of the complex.

In the presence of ligand-induced changes of the fluorescence intensity at 650 nm, Eq. (2) is replaced by

(3) $$R\left(c_{ligand}\right)=\frac{R_{unbound}+f\left(c_{ligand}\right)\cdot \left(R_{bound}\cdot r-R_{unbound}\right)}{1+f\left(c_{ligand}\right)\cdot \left(r-1\right)}$$

with

(4) $$r=\frac{F_{bound}}{F_{unbound}}$$

where F_bound_ and F_unbound_ are parameters obtained from fitting a sigmoidal dose–response curve to the fluorescence intensity at 650 nm. Ligand-induced changes of the fluorescence intensity at 670 nm have no effect on the ratiometric readout and therefore do not require a mathematical correction.

Van't Hoff plots were prepared by plotting the logarithm of the dissociation constant, ln K_d_, over the inverse temperature, 1/T. A linear regression was then used to calculate thermodynamic parameters according to

(5) ΔH=R⋅slope

and

(6) ΔS=−R⋅intercept

where R is the ideal gas constant.

## Results

### Characterizing Molecular Interactions with Spectral Shift

**Fig. 1.**
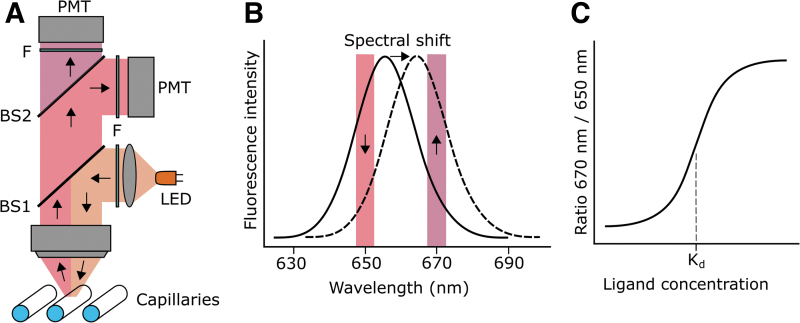
Experimental epifluorescence setup and spectral shift signal. **(A)** An amber LED produces excitation light of a peak wavelength of 592 nm that matches the secondary, shorter absorption peak of a suitable near-infrared fluorophore. The light is reflected on a beam splitter (BS1, 615 nm) and excites fluorescence within a capillary. The *red*-shifted emission then passes BS1 and is divided by a second beam splitter (BS2, 660 nm) into a lower and higher wavelength component. Filters (F) further clean up the emission light before being collected in PMTs. **(B)** *Red* dyes that undergo a spectral shift lead to a decrease of the 650 nm and an increase of the 670 nm fluorescence or vice versa. Note that filter widths are larger than depicted and, in fact, asymmetrical to maximize fluorescence collection. **(C)** Plotting the 670 nm/650 nm ratio against the logarithmic concentration of a non-fluorescent ligand leads to a sigmoidal binding curve that can be used to extract the K_d_ value of the interaction. LED, light-emitting diode; PMT, photon-multiplier-tube.

To develop a dual-emission epifluorescence setup, we focused on near-infrared fluorophores with an excitation maximum at around 660 nm. As illustrated in *Figure 1A*, an amber light-emitting diode is used to excite the fluorescently labeled molecules within capillaries. The emitted fluorescence is then split into a lower- and higher-wavelength component and further cleaned up by emission filters at 650 and 670 nm before reaching photo-multiplier tubes. Small hypsochromic (blue-) or bathochromic (red-) shifts of the emission peak of the fluorescent dye translate into large changes of the 670 nm/650 nm ratio (*Fig. 1B*).

Dissociation constants (K_d_) between a fluorescently labeled target and a non-fluorescent ligand molecule can be obtained with titration. In brief, equal amounts of target are mixed with a dilution series of ligand, and the ratio of the recorded fluorescence intensities at 670 and 650 nm are plotted against the logarithmic ligand concentration. The K_d_ can then be determined from the obtained sigmoidal binding curve (*Fig. 1C*).

### Protein–Small Molecule Interactions

One of the main aims in drug discovery is to identify small molecules that directly bind to and inhibit or modulate the function of a target protein. Therefore, we initially chose a small molecule–protein interaction to evaluate the ratiometric dual-emission approach. Importantly, we wanted to verify that ligand binding can be measured even in the absence of conformational changes and without the need for elaborate site-specific labeling techniques. We hence selected a very rigid protein that is known not to undergo any observable (>1 Å) conformational change upon small molecule binding: the enzyme carbonic anhydrase II.^31^ Carbonic anhydrase inhibitors have been well characterized by surface plasmon resonance (SPR) and isothermal titration calorimetry (ITC) and K_d_ values are readily available from literature.^32–34^

**Fig. 2.**
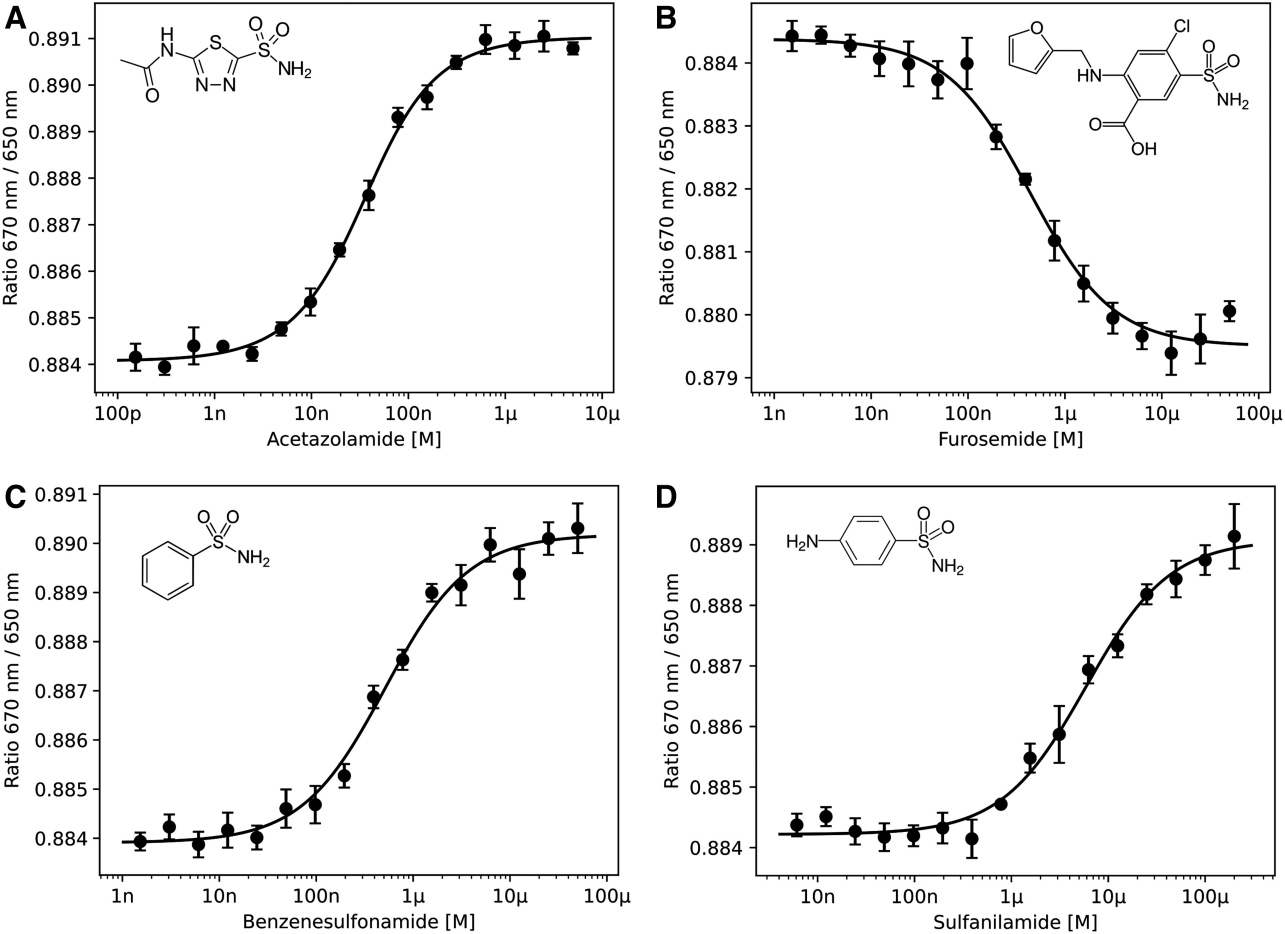
Spectral shift dose–response curves for four different bCA-II inhibitors: **(A)** acetazolamide, **(B)** furosemide, **(C)** benzenesulfonamide, and **(D)** sulfanilamide. Molecular structures of the inhibitors are displayed in the upper corners of each graph. Error bars represent standard error of *n* = 4 values. bCA-II, bovine carbonic anhydrase II.

Bovine carbonic anhydrase II (bCA-II) was fluorescently labeled using the 2nd Generation environment-sensitive dye, RED-NHS in the Protein Labeling Kit (cat# MO-L011; NanoTemper Technologies GmbH), which results in covalent labeling of lysine residues on the protein surface. bCA-II was then incubated with dilution series of the small molecule inhibitors acetazolamide, furosemide, benzenesulfonamide, or sulfanilamide and binding affinities were determined by spectral shift analysis (*Fig. 2*). The obtained K_d_ values show excellent correlation with published SPR and ITC data for all four inhibitors (*Table 2*).

**Table 2.** List of K_d_ Values Between bCA-II and Inhibitors

Inhibitor	K_d_ (spectral shift)	K_d_ (SPR)	K_d_ (ITC)
Acetazolamide	25.3 ± 2.3 nM	19 nM^32^	97 nM^33^
Furosemide	426 ± 63 nM	513 nM^32^	526 nM^33^
Benzenesulfonamide	502 ± 67 nM	848 nM^32^	839 nM^34^
Sulfonilamide	5.85 ± 0.73 μM	5.88 μM^32^	3.23 μM^33^

K_d_ values between bCA-II and four inhibitors obtained from spectral shift measurements, compared with literature results from SPR and ITC.

bCA-II, bovine carbonic anhydrase II; ITC, isothermal titration calorimetry; SPR, surface plasmon resonance.

Fluorescently labeled bCA-II without bound inhibitor exhibited a highly reproducible 670 nm/650 nm ratio of 0.884 in all assays. Upon binding of acetazolamide, the ratio increased to a value of 0.891, a relative change of less than 1%. Bathochromic shifts for benzenesulfonamide and sulfanilamide were found to be slightly smaller (final values of 0.890 and 0.889, respectively), while notably the ratio for furosemide changed to lower values. These very small differences in the emission spectra could not be resolved using a fluorescence spectrophotometer or fluorescence microplate reader (*Supplementary Fig. S1*).

### Membrane Protein–Ligand Interactions

Membrane proteins play crucial physiological roles *in vivo* and are a major class of drug targets. Here, spectral shift analysis was further evaluated by measuring and quantifying the interaction between the small molecule inhibitor BAY-876 and the GLUT1 transmembrane protein, which is a promising drug target for cancer treatment.^35^ Due to the presence of a C-terminal His_9_-tag, DDM-solubilized GLUT1 can be labeled with the Protein Labeling Kit RED-tris-NTA 2nd Generation (cat# MO-L018; NanoTemper Technologies GmbH). Non-covalent labeling has the advantage of being less disruptive to protein structure and is often the labeling strategy of choice for unstable or challenging targets such as membrane proteins.^36^

**Fig. 3.**
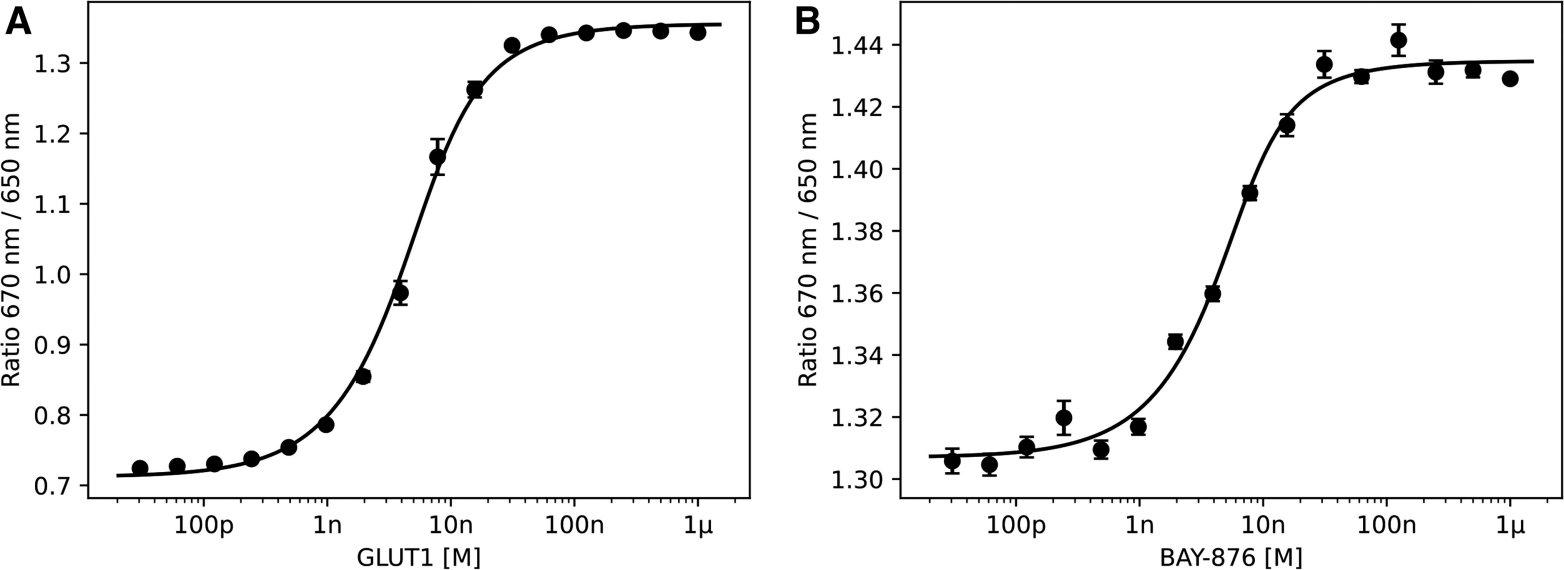
Spectral shift dose–response curves for the membrane transporter protein GLUT1. **(A)** Dose–response curve between the His_9_-tagged GLUT1 and the tris-NTA dye revealing a K_d_ value of 2.1 ± 0.2 nM. **(B)** Binding of the small molecule BAY-876 to GLUT1. Upon binding, the ratio further increased from ∼1.31 to ∼1.43. The K_d_ for the interaction was determined as 1.6 ± 0.4 nM. Error bars represent standard error of *n* = 4 values. GLUT1, glucose transporter 1.

As a first step, we probed the affinity of the His-tag for the tris-NTA dye (*Fig. 3A*). The accessibility of His-tags can vary significantly, and affinities between tris-NTA and a His-tag can range from high picomolar to high nanomolar values. It is good practice to calculate this affinity before conducting ligand-binding experiments. Binding of the tris-NTA dye to the His-tag resulted in a very large bathochromic shift, which almost doubled the 670 nm/650 nm ratio. The affinity between His-tag and dye was found to be ∼2 nM. We then mixed a dilution series of the small molecule BAY-876 with a final concentration of 20 nM labeled GLUT1 (*Fig. 3B*).

The interaction between GLUT1 and this highly selective inhibitor has been characterized previously and was shown to have an IC_50_ of 2 nM.^37^ Upon binding, the 670 nm/650 nm ratio displayed an even larger red-shift. The dose–response curve revealed very tight affinity with a K_d_ value of 1.6 ± 0.4 nM, which is in excellent agreement with the reported IC_50_ from cellular assays. To rule out that the spectral shift is due to a solvatochromic effect of BAY-876 directly interacting with the fluorophore, a dilution series of BAY-876 and tris-NTA dye in the absence of GLUT1 was prepared. In this control experiment, the 670 nm/650 nm ratio did not change (*Supplementary Fig. S2*).

### Antibody–Protein Interactions

Intrinsically disordered proteins (IDPs) are notoriously challenging to study. The structure of IDPs cannot be represented by a single conformation but must instead be described as an ensemble of interconverting conformations. One such IDP that is involved in neurodegeneration is tau.^38^ Tau is a microtubule-associated protein—abundant in neurons, where it promotes the assembly and maintains the structure of microtubules. It is also found in neurofibrillary tangles in Alzheimer's disease. Antibodies against various sites of tau are widely used in scientific research and are also being studied in clinical assays.^39^ Thus, it is important to know the specificity and selectivity of antibodies to tau epitopes in different tau species (monomeric full length tau isoforms and truncated tau fragments, oligomers, and fibrils) and to characterize tau epitopes more closely.

We asked whether the protein–protein interaction between the anti-tau antibody Tau5 and several isoforms and fragments of tau could be monitored using spectral shift analysis. For this, we covalently labeled lysine residues of Tau5 using the Protein Labeling Kit RED-NHS 2nd Generation (cat# MO-L011; NanoTemper Technologies GmbH). As ligands, the two tau isoforms 2N4R (tau-441) and 0N3R (tau-352), and the shorter tau fragments tau_221–441_ and tau_343–441_ were selected. It has been shown previously that Tau5 binds to the Pro218–Lys225 epitope of tau and the structure of the complex is known.^40–42^ Therefore, the antibody should bind to both isoforms but not to the two shorter fragments.

**Fig. 4.**
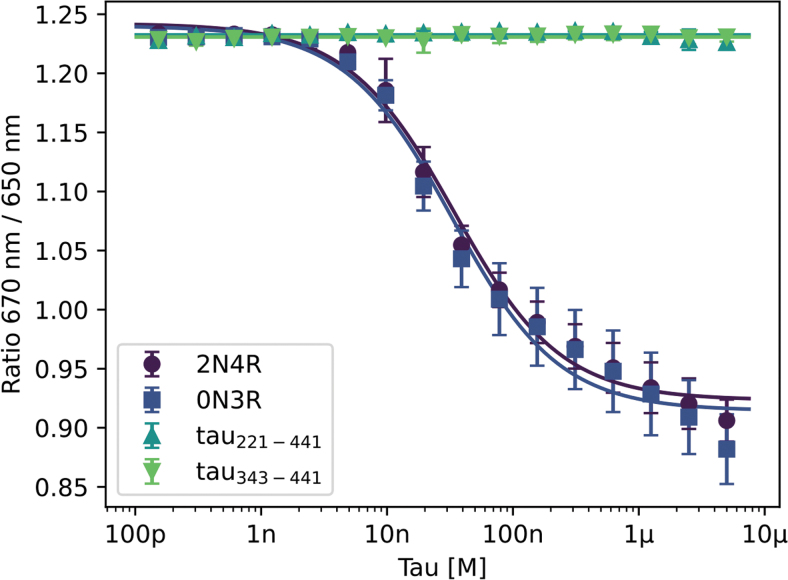
Binding of fluorescently labeled Tau5 antibody to several tau isoforms and fragments. Spectral shift dose–response curves show that Tau5 binds to tau isoforms 2N4R and 0N3R with affinities of 29.8 ± 3.6 nM and 28.7 ± 4.8 nM, while it does not bind to the shorter fragments tau_221–441_ and tau_343–441_. Error bars represent standard error of *n* = 5 values.

Indeed, only 2N4R and 0N3R were recognized by Tau5, resulting in K_d_ values of 29.8 ± 3.6 nM and 28.7 ± 4.8 nM, respectively. The two shorter fragments that either lack the full epitope or contain only a part of it (tau_343–441_ and tau_221–441_, respectively) did not show any binding (*Fig. 4*), which further defines the tau epitope to which the antibody binds.

It is important to note that when measuring antibody binding to surface-immobilized ligands, avidity effects can lead to exaggerated low picomolar binding affinities, when suboptimal experimental design is used.^39^ Also, the negative charge of the CM5 sensor chip most frequently used for SPR measurements can introduce artifacts for the interactions of positively charged tau proteins.^43^ This can be prevented by applying an in-solution methodology. Indeed, the obtained affinities by spectral shift analysis lie in the same low to high nanomolar range as monovalent affinities determined with SPR for several clinical anti-tau antibodies against monomeric full-length tau.^44^

### Thermodynamics of DNA Hybridization

A molecular interaction is often solely described by its dissociation constant K_d_. However, valuable information about the interaction can also be revealed by its thermodynamic properties, such as the enthalpy and entropy of binding. As a general paradigm in drug development, enthalpy-driven binding is believed to result in more effective drugs with superior physicochemical properties than entropy-driven binding, as it indicates specific interactions between target and ligand.^45^ A popular strategy to derive thermodynamic parameters for an interaction is the so-called van't Hoff analysis, in which dissociation constants are determined at multiple temperatures instead of only a single one.^46^

**Fig. 5.**
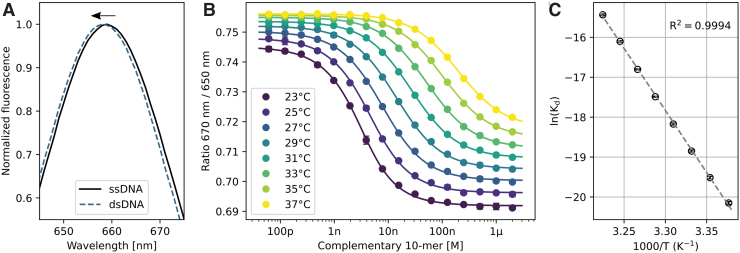
Thermodynamics of DNA hybridization. **(A)** Full emission spectra measurements of Cy5-labeled 13-mer alone (ssDNA, 1 μM) and mixed with an excess of its complementary 10-mer sequence (dsDNA, 2 μM) using a fluorescence spectrometer. Upon hybridization, the Cy5 emission shows a small hypsochromic shift, indicated by the *black arrow*. **(B)** Dose–response curves of hybridization measurements between 2.5 nM Cy5-labeled 13-mer and a dilution series of complementary 10-mer at eight different temperatures between 23°C and 37°C, recorded with the dual-emission setup described in this work. Error bars represent standard error of *n* = 2 values from a heating and subsequent cooling cycle. Dose–response curves shift toward higher concentrations of complementary DNA for higher temperatures, which shows that binding is enthalpically driven. **(C)** Van't Hoff analysis of the DNA hybridization reaction. Enthalpy and entropy values of the interaction were obtained by plotting the logarithm of the dissociation constant K_d_ versus the inverse of the applied temperature, revealing a linear relationship (*R*^2^ = 0.9994) and thermodynamic parameters of ΔH = −61.8 kcal/mol and ΔS = −168.5 cal/mol/K.

To assess whether the presented method can also be used for the thermodynamic characterization of an interaction, we selected a hybridization reaction between a 13-mer Cy5-labeled target strand and a complementary 10-mer. To prevent stabilizing effects of the Cy5 dye on the DNA duplex, a short poly-T spacer was inserted between dye and target sequence.^47^ Initial full-spectrum measurements of the Cy5-labeled strand with a fluorescence spectrometer confirmed that the Cy5 dye experiences a hypsochromic shift of approximately one nanometer upon duplex formation (*Fig. 5A*). Ratiometric fluorescence measurements with the dual-emission setup described here were carried out in 2°C increments at eight different temperatures between 23°C and 37°C (*Fig. 5B*).

In agreement with the full-spectrum measurements, the 670 nm/650 nm ratio decreased upon hybridization. The K_d_ of the hybridization reaction was found to be highly temperature-dependent and approximately doubled with each 2°C temperature increment (*Table 3*). Equilibrium conditions were confirmed from the superposition of dose–response curves from heating and cooling cycles. The thermodynamic analysis yielded a van't Hoff enthalpy of ΔH = −61.8 kcal/mol and entropy of ΔS = −168.5 cal/mol/K (*Fig. 5C*). The observed thermodynamic parameters are in good agreement with published values for oligonucleotides of a similar sequence.^47,48^

**Table 3.** Effect of Temperature on DNA Hybridization

T (°C)	23	25	27	29	31	33	35	37
K_d_ (nM)	1.77 ± 0.06	3.35 ± 0.11	6.52 ± 0.17	12.8 ± 0.3	25.4 ± 0.4	50.4 ± 0.7	102 ± 2	198 ± 4

Overview of K_d_ values between Cy5-labeled 13-mer (5′ Cy5 TTT GGA CTT CAG G 3′) and a complementary 10-mer (3′ CCT GAA GTC C 5′), determined from spectral shift analysis.

## Discussion

Isothermal spectral shift analysis is based on the phenomenon that not only peak intensities but also wavelength maxima of fluorophores attached to a target molecule can change when their chemical microenvironment is altered. The cause of the shift may be a conformational change of the target upon ligand binding or simply the presence of the ligand in close proximity to the dye. By detecting the emission fluorescence simultaneously at two separate wavelengths, even the smallest bathochromic or hypsochromic shifts of the emission peak can result in detectable ratiometric changes. The method is analogous to the intrinsic protein fluorescence measurements used in nano differential scanning fluorimetry, in which protein unfolding is monitored with high sensitivity and precision by observing changes in the 350 nm/330 nm ratio of tryptophan and tyrosine fluorescence.^49^

Tryptophan often undergoes large spectral shifts when its microenvironment turns from hydrophobic to hydrophilic during protein denaturation. The effect, however, is not exclusively limited to protein unfolding but has also been used to monitor ligand binding.^4^ However, since autofluorescence in the ultraviolet range is very widespread, the adoption of near-infrared dyes offers the advantage of much lower interference and background signal. It thus extends the range of application to measurements of, for example, protein–protein interactions and autofluorescent low-molecular-weight ligands.

Since shifts of absorbance and emission peaks are often correlated, ratiometric readings can, in principle, also be obtained by exciting the dye at two different wavelengths instead of detecting the fluorescence with dual-emission optics. However, while technically feasible, the dual-excitation configuration poses several challenges.

First and foremost, two excitations cannot be measured simultaneously, which means the data would need to be acquired sequentially. Not only is this more time-consuming, but the time delay between measurements can also introduce differences in the state of the sample between measurements, for example, due to bleaching or time-dependent adsorption or aggregation. Second, while many dyes can also be excited efficiently at a wavelength slightly below the wavelength of their maximum absorbance, emission spectra tend to show only a single dominant peak. For fluorophores with small Stokes shifts, it can therefore become difficult to separate excitation and emission well enough. Simultaneous fluorescence acquisition in a dual emission setup avoids both of these problems.

Ratiometric fluorescence measurements of bCA-II and its inhibitors showed that spectral shifts do not have to be large to be detectable. We estimate that the observed signal changes correspond to shifts in the maximum fluorescence wavelength of less than one hundred picometers—too low to be measured with a conventional plate reader or fluorimeter. This can be explained by the high rigidity of bCA-II, which is known to not undergo large conformational rearrangements upon binding. The dominating effect causing the spectral shift must therefore be due to smaller structural changes occurring in the proximity of the ligand binding site. Upon examining the chemical structures of the four evaluated small molecules, only furosemide possesses a furan moiety that protrudes out of the active site and can potentially come into contact with the fluorescent dye.

This may explain the observation that furosemide was also the only inhibitor that caused a blue-shift instead of a red-shift upon binding. The effect on binding affinity is negligible as the affinity measured is well within the range measured with SPR and ITC (*Table 2*). Regarding the small magnitude of the observed spectral shift, it also becomes clear that the environment-sensitivity of the dye is a crucial factor for the success of the method. Since spectral shifts and quenching effects are often undesired in many other applications like FRET, where they would interfere with the measured efficiencies, many commercially available dyes have been optimized to be unaffected by changes in their local environment. Those fluorophores are therefore not suited for spectral shift measurements.

By characterizing the interaction between His-tagged GLUT1 and its small molecule inhibitor BAY-876, we showed that the described method is sensitive enough to precisely quantify an interaction even if the label is distant from the binding site. This was also confirmed by measuring protein–protein interactions using a labeled antibody. Since lysine residues are predominantly found in the Fc chain of antibodies, we assume that the fluorescent label is likely attached in that region and thus located relatively far from the antibody binding region on the Fab segment. Despite this, large ratiometric signal changes were still observed upon antigen binding, again highlighting the sensitivity of this method.

The ratiometric dual-emission approach is ideally suited for measurements at different temperatures since the same samples can be used for each temperature measurement, which tremendously reduces sample consumption and time. Sealing of the capillaries is recommended when a large temperature range is probed to prevent evaporation and thus concentration changes within the dilution series, which could otherwise affect the resulting K_d_. An important factor in these experiments is also to ensure that the protein is stable within the selected temperature range.

When preparing ligand solutions for a spectral shift measurement, it is crucial that the buffer composition does not change throughout the dilution series. Red dyes have been found to experience red- or blue-shifts in presence of glycerol or detergent that can create spectral shift signals similar to a ligand-binding event.^24^ The same holds true for differences in solvent polarity that can occur due to differences in DMSO concentration.

As with fluorescence polarization assays, artifacts from concentration-response measurements can also occur when ligands absorb excitation or emission light. This is especially noteworthy since fluorescently labeled targets are usually used at low nanomolar concentrations, while small molecule ligands are typically tested in the micromolar range. Control experiments using a dilution series of ligand with a non-binding fluorescent target or dye alone can rule out such false positives. Finally, as with any method that involves a fluorescently labeled or surface-immobilized target, it is imperative to ensure that the covalent modification does not alter the binding interaction. Fluorophores that block the binding site of a protein target can impair or even completely abolish an interaction. In those cases, an alternative labeling strategy needs to be pursued.

## Conclusions

This work shows that isothermal spectral shift analysis can resolve even sub-nanometer spectral shifts of the emission wavelength maximum of a fluorescently labeled target to derive binding affinities with high precision. Low nanomolar concentrations of fluorescent molecules are sufficient, which lies outside the typical sensitivity and detection range of conventional fluorimeters or plate readers. In addition, molecular interactions can be characterized without the need for conformational changes upon ligand binding or site-specific labeling of the target molecule. This allows for broad applicability of the described method, turning it into a valuable technique well suited for analyzing challenging biomolecular interactions.

Finally, the ease and speed of performing ratiometric measurements at different temperatures, paired with the very low sample consumption, makes it also an attractive tool for determining thermodynamic parameters such as binding enthalpies and entropies.

## Supplementary Material

Supplemental data

Supplemental data

## Abbreviations Used

bCA-II: bovine carbonic anhydrase II
BRET: bioluminescence resonance energy transfer
DMSO: dimethyl sulfoxide
FP: fluorescence polarization/anisotropy
FRET: fluorescence resonance energy transfer
GLUT1: glucose transporter 1
IDP: intrinsically disordered protein
ITC: isothermal titration calorimetry
LED: light-emitting diode
PBS: phosphate-buffered saline
PMT: photon-multiplier-tube
SPR: surface plasmon resonance
