# Supplementary material for: A New Spectral Shift-Based Method to Characterize Molecular Interactions
Source: Assay Drug Dev Technol. 2022 Mar 8;20(2):83–94. doi: 10.1089/adt.2021.133 (PMC8968852; doi:10.1089/adt.2021.133)
Supplement: Supplemental data [file Suppl_FigS1.docx]

SUPPLEMENTARY DATA

| 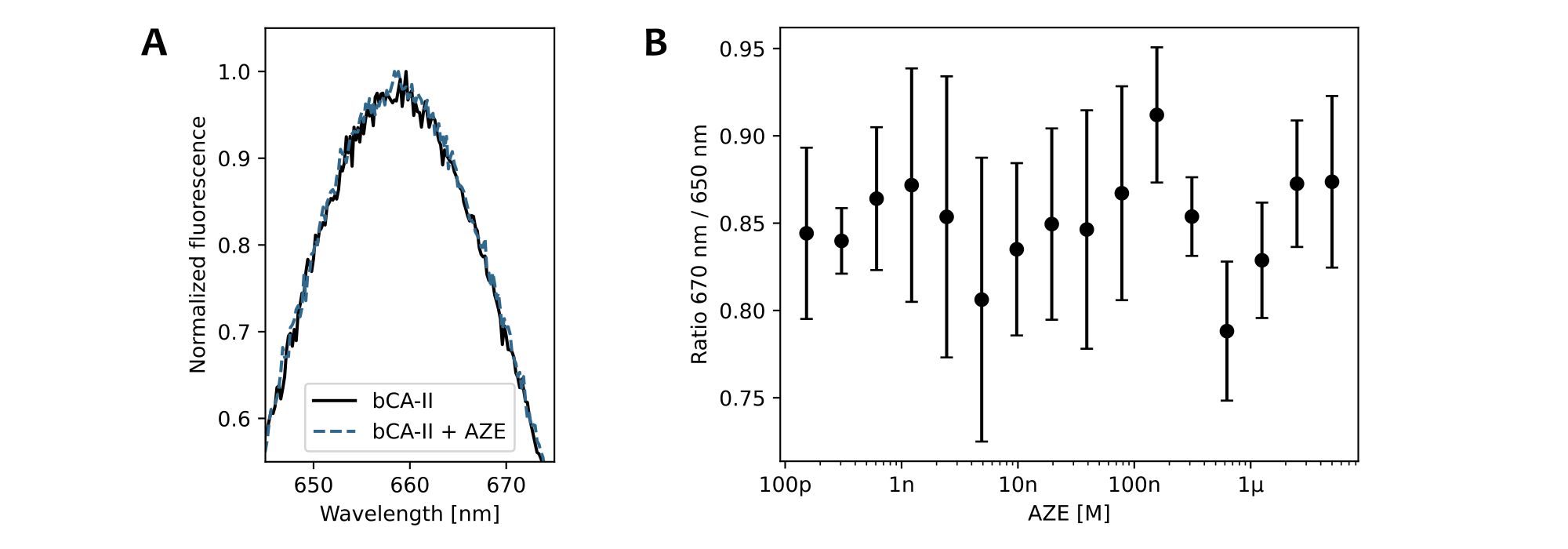 |
| --- |

**Fig. S1.** Fluorescence spectrophotometer and fluorescence microplate reader measurements. (**A**) Full emission spectra measurements of fluorescently labeled carbonic anhydrase (bCA-II, 20 nM) mixed with an excess of acetazolamide (AZE, 1 µM) using a fluorescence spectrometer. The noise in the spectra is too high to observe any spectral shift. (**B**) Measurement of a dilution series of AZE against labeled bCA-II (20 nM) in a fluorescence microplate reader. Error bars represent standard error of n=3 values. At the low concentration of fluorescent target that is needed to accurately quantify the K_d_ between bCA-II and AZE, the noise in the data is too large to observe an interaction.
