# Supplementary material for: A New Spectral Shift-Based Method to Characterize Molecular Interactions
Source: Assay Drug Dev Technol. 2022 Mar 8;20(2):83–94. doi: 10.1089/adt.2021.133 (PMC8968852; doi:10.1089/adt.2021.133)
Supplement: Supplemental data [file Suppl_FigS2.docx]

| 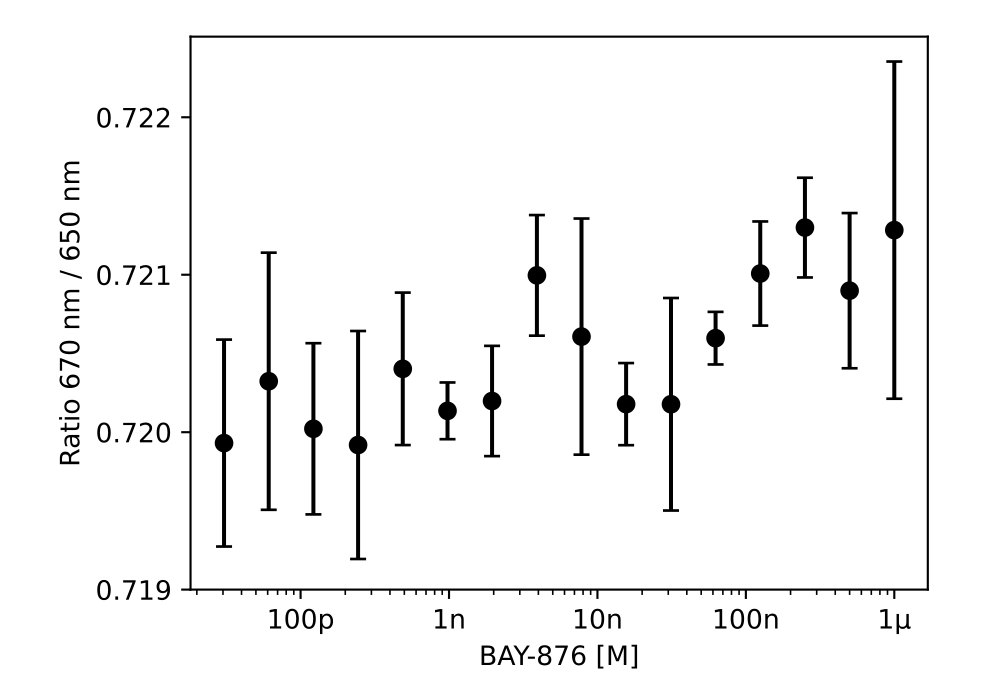 |
| --- |

**Fig. S2.** Control experiment for GLUT1. Dose response curve between RED-tris-NTA dye and BAY-876 in the absence of His-tagged GLUT1, measured with Monolith X. BAY-876 alone does not alter the 670 nm / 650 nm ratio and a solvatochromic effect can be excluded. Error bars represent standard error of n=4 values.
